# Management of Non-High-Risk Salivary Gland Carcinomas With Surgery Alone

**DOI:** 10.7759/cureus.16970

**Published:** 2021-08-07

**Authors:** Sepehr Shabani, Abhay V Sharma, Matthew L Carmichael, Tapan A Padhya, Matthew J Mifsud

**Affiliations:** 1 Otolaryngology - Head and Neck Surgery, University of South Florida Morsani College of Medicine, Tampa, USA

**Keywords:** head and neck tumors, parotid tumor, submandibular neoplasm, adjuvant radiation therapy, malignancy surgery

## Abstract

Background

Risk stratification and appropriate treatment selection are essential for the management of head and neck malignancies, in order to optimize long-term outcomes. Salivary gland carcinomas (SGCs) pose a particular challenge due to their extensive biologic heterogeneity. Primary surgical resection remains the mainstay of treatment; however, outcomes with single modality therapy for 'non-high-risk' lesions are less elucidated in the literature present on the subject. We present our experience with non-high-risk salivary gland malignancies treated by surgery alone.

Methods

A retrospective analysis of SGCs from 1998-2011 was completed after receiving Institutional Review Board approval. Patient demographic, tumor, treatment, and outcome data were obtained from chart review. The primary outcomes of interest were overall survival (OS) and recurrence-free survival (RFS).

Results

Of the 62 patients identified, 49 patients underwent resection of the primary tumor alone, while an ipsilateral selective neck dissection was included for 13 patients. The median follow-up was 5.05 years. Of the tumors, 79% were low-intermediate grade, 3% high grade, and 17% poorly classified. The OS and RFS were 91% and 87% at five years and 80% and 79% at 10 years, respectively. The combined failure rate of local, regional, and distance was 13%.

Conclusion

Surgery alone is an appropriate treatment strategy for patients with non-high-risk salivary gland malignancy, affording a high likelihood of long-term RFS and OS.

## Introduction

Salivary gland malignancies are a heterogenous group of neoplasms, with more than 24 different disease types categorized [1]. They are also relatively rare, a cause of only 3-7% of head and neck cancers in the USA annually [2, 3]. This rarity and histologic diversity pose an extensive clinical challenge, preventing the development of well-established/evidence-based treatment algorithms to best optimize treatment decisions. Nevertheless, surgical resection is an essential component to the primary treatment of these neoplasms when feasible [4, 5]. Nonsurgical treatment strategies have typically been associated with decreased tumor control rates, often associated with a presumption of radio-resistance for the majority of salivary gland cancers (SGCs) [3, 5-8].

In addition to the standard American Joint Committee on Cancer (AJCC) tumor, nodes, and metastases (TNM) staging system, key clinicopathologic variables are often considered for risk stratification. Pioneering work by Foote and Frazell in the 1950s, for example, first linked distinct tumor histologies with unique disease phenotypes [4]. As our understanding of this collection of malignancies expands, prognostication schemes continue to evolve. Certain features, however, are almost universally accepted to be associated with aggressive clinical behavior, in particular, high-grade histology (if well defined), gross 'named nerve' invasion, advanced tumor size (>4cm), positive surgical margins, and cervical lymph node spread [1, 9, 10]. A multi-modality approach is the typical standard for these high-risk cases, with the addition of adjuvant radiotherapy (after surgery) affording enhanced disease control [3, 11]. Terhaard et al, for example, reported significant improvement in 10-year local disease control for advanced (T3-T4) cases when adjuvant radiotherapy was utilized compared to surgery alone (84% vs 18%) [12].

Much published research has similarly concentrated on high-risk SGCs, with non-high-risk cases often included or combined into large series as a relative afterthought. We thus present our own experience with non-high-risk salivary malignancies (non-angioinvasive, non-infiltrative) [13], managed with surgery alone. In reviewing the long-term results of our data, we hope to demonstrate the acceptable application of this management strategy. 

This article was previously presented as meeting abstracts at two conferences (one national and one regional): the 2019 Combined Otolaryngology Spring Meeting-AHNS on May 1, 2019, and the 2019 Florida Combined Otolaryngology Meeting on November 8, 2019.

## Materials and methods

This study was approved by the Institutional Review Board of H. Lee Moffitt Cancer Center and Research Institute (Pro0000923). A retrospective chart review was performed on patients with either primary major or minor SGCs managed between 1998 and 2011. The year 2011 was chosen as the cutoff year to allow long-term follow-up, given that late recurrence is a common feature of many salivary malignancies. Chart review was performed and completed in June 2019.

Study inclusions were as followed: single modality surgical therapy (tumor resection ± neck dissection) of the initial tumor with curative intent, absence of cervical lymph node spread (cN0 or pN0), no evidence of disease metastasis (M0), and a negative surgical margin (R0) after final pathological assessment. Squamous cell carcinomas arising within major salivary glands were excluded given the difficulty distinguishing this potential entity from metastatic cutaneous carcinomas. After identification of patients, the following information was gathered: demographics, age at diagnosis, date of surgery, tumor characteristics, status and date of locoregional recurrence, status and date of distance metastasis, date of last follow-up, and date of death (if appropriate). All cases were uniformly staged using AJCC seventh edition TNM guidelines. 

Endpoints of interest were overall survival (OS) calculated from the date of surgery to the final patient contact or mortality and recurrence-free survival (RFS) calculated from the date of surgery to final follow-up with detail of disease status. All statistical analyses were performed using the Statistical Package for the Social Sciences (SPSS) Statistics Version 25.0 (IBM, Armonk, NY). Survival data was reported utilizing the Kaplan-Meier method. Comparisons were made with a log-rank test with a p-value < 0.05 considered for significance.

## Results

After query of institutional data, 63 patients initially met inclusion criteria for this review; a single patient was, however, excluded due to a suicide on post-op day two, leaving a total of 62 cases. Patient and disease characteristics are listed in Table 1. Of the selected patients, 34 (55%) were female and 28 (45%) were male. The median age at diagnosis was 60 years with females presenting at a younger age compared to males (57 vs. 60). Forty-nine patients underwent resection of the primary tumor alone, while an ipsilateral selective neck dissection was included for 13 patients who invoked a concern for high-grade tumors on fine-needle aspiration/intra-operative frozen section or cervical lymphadenopathy on pre-operative imaging. The median follow-up was 5.05 years.

**Table 1:** Demographic, tumor, and clinical characteristics for patients with malignant salivary tumors. (n=62)

Median Age (Years)	Female	57	
	Male	60	
		Number of Patients	Percentage of Patients (%)
Gender	Female	34	55
	Male	28	45
Tumor Site	Major Salivary Glands	17	27
	Parotid	14	23
	Submandibular	3	5
	Minor Salivary Glands	45	73
	Oral cavity	30	48
	Oropharynx	10	16
	Sinonasal	4	6
	Larynx	1	2
Tumor Grade	Low-Intermediate	49	79
	Poorly classified	11	18
	High	2	3
Histology	Mucoepidermoid carcinoma	26	42
	Adenoid cystic carcinoma	11	18
	Acinic cell carcinoma	10	16
	Polymorphous low-grade adenocarcinoma	5	8
	Adenocarcinoma	4	6
	Carcinoma ex-pleomorphic adenoma	2	3
	Oncocytic carcinoma	1	2
	Salivary duct carcinoma	1	2
	Basal Cell adenocarcinoma	1	2
	Adenosquamous carcinoma	1	2
T-Classification	1	49	79
	2	8	13
	3	5	8

There was a preponderance of minor SGCs, which comprised 73% of all cases. As would be expected, these were most commonly localized within the oral cavity (n=30), particularly common at the hard/soft palate junction. The major salivary gland cases were mainly localized to the parotid gland (n=14) with a small cohort of submandibular (n=3) cancers. The histologic distribution was similar to convention, with the most common tumor types being mucoepidermoid carcinoma (42%), adenoid cystic carcinoma (18%), and adenocarcinomas (16%). Of the cases, 92% were staged T1-T2 and 79% were classified as being low to intermediate grade.

**Figure 1:**
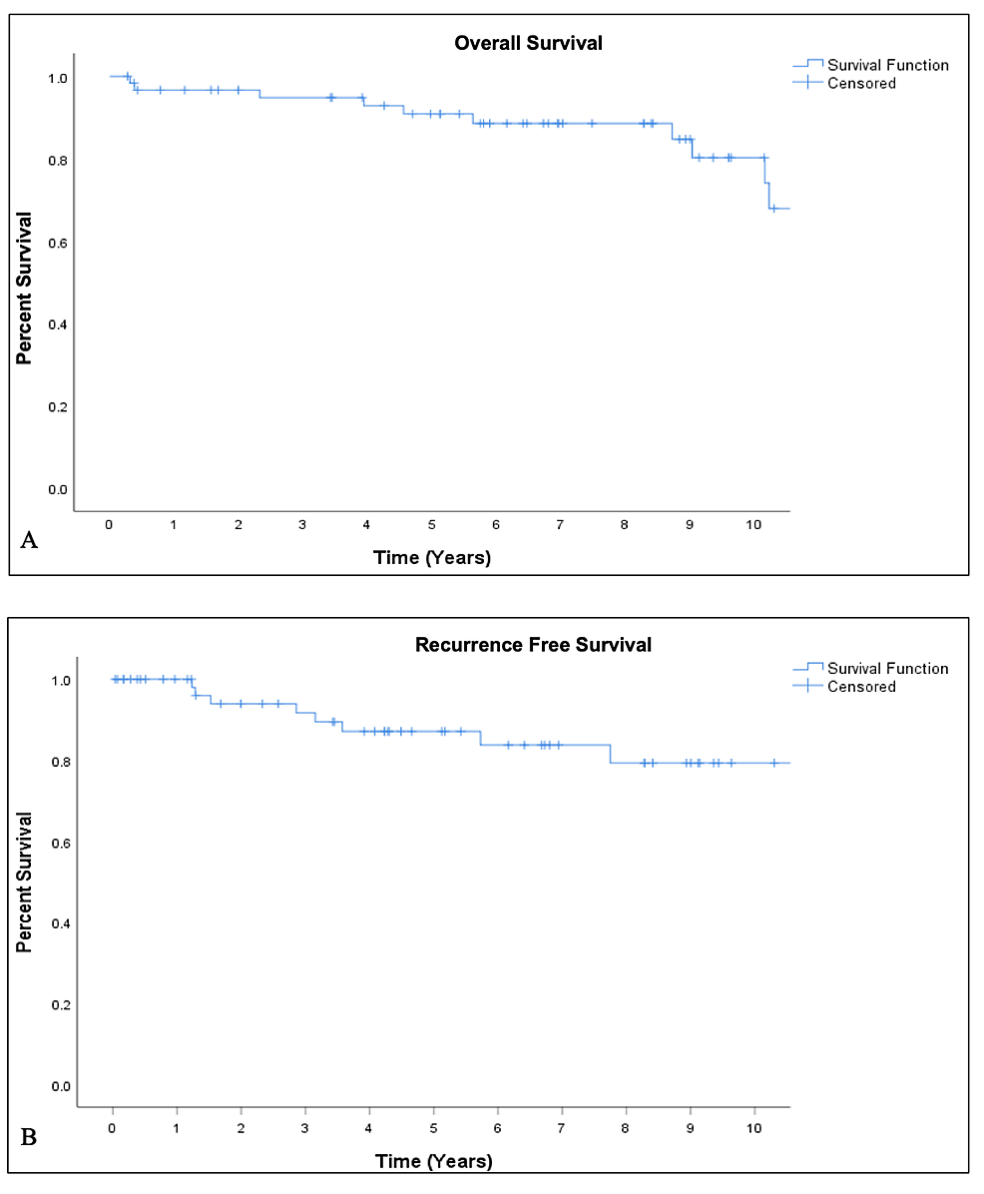
Kaplan-Meier curves for overall survival (A) and recurrence-free survival (B)

Figure 1 demonstrates overall treatment outcomes for this group. The OS and RFS were 91% and 87% at five years and 80% and 79% at 10 years, respectively. For sub-group analyses, five-year rates were calculated. When comparing females to males, both five-year OS (93% vs. 88%, p = 0.61) and RSS (82% vs. 78%, p=0.815) were higher, although this did not meet statistical significance. T-stage corresponded well with the likelihood of disease recurrence, with five-year RFS for T1, T2, and T3 tumors of 92%, 80%, and 50%, respectively (p = 0.042). When stratified for tumor site, five-year RFS was highest for oral cavity cancers (96%) and lowest for cancers arising within the sinonasal tract (50%); however, given the low numbers, the statistical significance of this finding cannot be well defined. 

During the follow-up period, there were a total of 12 episodes of disease recurrence, including a single case of concurrent local + regional failure, occurring in a total of eight patients (Table 2). Characteristics of all eight patients are documented in Table 3. Local, regional, and distant recurrence rates are 10% (6/62), 3% (2/62) and 5% (3/62) respectively. The average time to first treatment failure was 3.87 years. Interestingly, locoregional failures seem to occur at an earlier average time course than distant disease metastases (3.1 vs 11.3 years). 

**Table 2:** Treatment failure characteristics

Failure Type	Episodes of Failure	Average Time (years)
Any	12	6.34
First Failure	9	3.87
Local	7	4.77
Regional	2	4.49
Distant	3	11.25

**Table 3:** Demographic, tumor, and clinical characteristics for patients with local, regional, or distance failure (n=8) † Patients with multiple failures ‡ Simultaneous recurrence; however, considered as two separate events for analysis

	Age	Gender	Tumor Site	Tumor Grade	Histology	T-stage	Failure Pattern	Failure from Surgery (years)	Neck Dissection
Major Salivary Gland	60	Female	Parotid	Mucoepidermoid Carcinoma	Low-intermediate	1	Local	3.15	No
	62	Female	Parotid	Acinic Cell Carcinoma	Low-intermediate	2	Local	2.86	No
	60†	Male	Submandibular	Oncocytic Carcinoma	High	1	Locoregional ‡ Distant	7.75, 8.19	Yes
Minor Salivary Gland	65	Male	Oral Cavity	Adenoid Cystic Carcinoma	Low-intermediate	1	Distant	5.73	Yes
	32†	Female	Oral Cavity	Adenoid Cystic Carcinoma	Low-intermediate	1	Local, Local, Distant	1.52, 13.23, 19.84	No
	75	Female	Sinonasal	Mucoepidermoid Carcinoma	Low-intermediate	1	Local	1.28	No
	70	Female	Sinonasal	Basal Cell Adenocarcinoma	Low-intermediate	3	Local	3.58	No
	72	Male	Oropharynx	Salivary Duct Carcinoma	Low-intermediate	3	Regional	1.23	No

## Discussion

Much treatment data on non-high-risk salivary malignancies has been derived from large population-based databases, e.g. the surveillance, epidemiology, and end results (SEER) data, with limited information on local/regional disease recurrence [14]. This cohort is thus a useful addition to the literature, as our patients were managed with a uniform treatment algorithm (surgery alone for low-intermediate tumor grade, pN0 or cN0, R0 resection, M0, no lymphovascular invasion, and infiltration) and have maintained prolonged follow-up. This is essential given a known propensity of SGCs for late recurrence. Chen et al., for example, report a cumulative probability of recurrence at 10 and 15 years after initial SGC treatment to be 13% and 18% respectively. Disease-free survival in their population thus slowly declines for at least 15-20 years from the date of initial surgery before reaching a plateau [15]. Park et al., confirmed this finding, as they report a median time to recurrence of 7.71 years after treatment, in their cohort of 240 SGC cases [16]. Short-term disease outcomes must thus be looked at with caution when considering treatment approaches for this group of malignancies. 

Our treatment results with surgery alone are comparable to series that have reported on multimodality therapy for non-high-risk salivary cancers. Armstrong et al., for example, noted five-year survival rates of 82% for 46 patients with non-metastatic T1 or T2 tumors of major salivary gland treated with combined surgery and postoperative radiotherapy [17]. A similar study by Zbaren et al. compared two patient cohorts with T1/T2 parotid malignancies and suggested an improvement in five-year RFS in those patients receiving adjuvant radiotherapy (92% vs. 70%). However, in this series, 40% of the surgery-only cohort were considered to have high-grade malignancies - which could explain the relatively high failure rate in this group [18]. 

Appropriate selection of patients with non-high-risk salivary carcinomas to receive surgery alone has the potential to both ensure comparable rates of disease control and avoid the potential adverse effects of adjuvant radiation therapy such as xerostomia, reduced taste, and lymphedema/post-radiation fibrosis. Becker et al. for example evaluated post-therapeutic health-related quality of life in patients with major SGCs treated with various management schemes [19]. Those patients requiring adjuvant radiotherapy had the most dramatic decline in quality of life, specifically negatively impacting physical appearance, activity, recreation, taste, and saliva [19]. In contrast, assuming facial nerve function is preserved, standard parotid surgery has been shown to have a very limited impact on long-term health-related quality of life after the recovery period [20, 21].

Our data should be considered favorably when compared to series that have managed SGCs with primary radiotherapy. For example, the University of California, San Francisco (UCSF) experience with primary radiotherapy for salivary malignancies noted five-year and 10-year OS rates of only 70% and 46% [6]. It should be mentioned that 27% of their patients had T4 disease, which is not comparable to our series. In a separate series of 67 patients by Holtzam et al., 10-year OS and local-regional control rates for stage I-III SGCs were 63% and 72% respectively after radiotherapy alone [22]. Primary surgical management not only provides superior disease control but facilitates pathologic assessment, which is essential for accurate prognostication and treatment planning. With that being said, radiotherapy is a reasonable alternative primary treatment, either for those deemed inappropriate surgical candidates (due to systemic medical comorbidities) or those who prefer non-surgical treatment (after a multidisciplinary treatment discussion) [7]. 

The above study must be considered in the setting of inherent limitations, specifically related to the inherent bias of retrospective data collection. We have also chosen to present a heterogeneous cohort of patients in regard to the tumor site. This could be potentially problematic, given the theoretical possibility of different disease phenotypes (despite otherwise identical histopathologic features) at separate locations. Tumor grade is another particular challenge in this population, as classification schemes may differ for certain tumor types, often without a clear consensus. This has also evolved somewhat even over the current study period. As a consequence, roughly 17% of cases were 'unclassified', with some of these entities potentially having high-risk features that would be less appropriate for single modality treatment.

## Conclusions

Salivary gland malignancies continue to pose a rare and particularly unique clinical challenge. Given the extensive heterogeneity at presentation, a variety of clinicopathologic risk variables must be considered in addition to the standard staging algorithms, in order to define the appropriate treatment approach. The goal of treatment optimization should not only focus on disease control but also on limiting post-treatment morbidity when appropriate. Based on our analysis, for appropriately selected low-risk SGCs, surgery alone can produce a high likelihood of long-term disease control.
